# The Effect of Host miRNAs on Prognosis in COVID-19: miRNA-155 May Promote Severity via Targeting Suppressor of Cytokine Signaling 1 (*SOCS1*) Gene

**DOI:** 10.3390/genes13071146

**Published:** 2022-06-25

**Authors:** Asuman Gedikbasi, Gokhan Adas, Nilgun Isiksacan, Kadriye Kart Yasar, Esra Canbolat Unlu, Rabia Yilmaz, Gulsum Oya Hergunsel, Zafer Cukurova

**Affiliations:** 1 Division of Medical Genetics, Department of Pediatric Basic Sciences, Institute of Child Health, Istanbul University, 34093 Istanbul, Turkey; 2 Department of Surgery, Bakirkoy Dr. Sadi Konuk Training and Research Hospital, University of Health Sciences, 34180 Istanbul, Turkey; gokhantolgaadas@gmail.com; 3 Stem Cell and Gene Therapies Application and Research Center, University of Health Sciences, 34668 Istanbul, Turkey; 4 Department of Biochemistry, Bakirkoy Dr. Sadi Konuk Training and Research Hospital, University of Health Sciences, 34180 Istanbul, Turkey; nisiksacan@gmail.com; 5 Department of Infectious Diseases and Clinical Microbiology, Bakırköy Dr. Sadi Konuk Training and Research Hospital, University of Health Sciences, 34180 Istanbul, Turkey; kadriye.kartyasar@sbu.edu.tr (K.K.Y.); canbolat-esra@hotmail.com (E.C.U.); 6 Department of Anesthesia and Reanimation, Bakırköy Dr. Sadi Konuk Training and Research Hospital, University of Health Sciences, 34180 Istanbul, Turkey; drrabiayilmaz@gmail.com (R.Y.); oyahergunsel@hotmail.com (G.O.H.); zcukurova@gmail.com (Z.C.)

**Keywords:** COVID-19, prognosis, miR-155-5p, suppressor of cytokine signaling 1 (*SOCS1*)

## Abstract

The epigenetic features contribute to variations in host susceptibility to SARS-CoV-2 infection and severity of symptoms. This study aimed to evaluate the relationship between the relative expression of microRNAs (miRNAs) and the severity of the disease in COVID-19 patients. The miRNA profiles were monitored during the different stages of the disease course using reverse transcription–quantitative polymerase chain reaction (RT-qPCR). The expression levels of the selected 11 miRNAs were measured in the blood samples collected from 73 patients (moderate, *n* = 37; severe, *n* = 25; critically ill, *n* = 11, a total of 219 longitudinal samples) on hospitalization day and days 7 and 21. Expression changes were expressed as “fold change” compared to healthy controls (*n* = 10). Our study found that several miRNAs differed according to disease severity, with the miR-155-5p the most strongly upregulated (*p* = 0.0001). A statistically significant negative correlation was observed between the expression of miR-155-5p and its target gene, the suppressor of cytokine signaling 1 (*SOCS1*). The relative expression of miR-155-5p was significantly increased and *SOCS1* was significantly decreased with the disease progression (r = −0.805 *p* = 0.0001, r = −0.940 *p* = 0.0001, r = −0.933 *p* = 0.0001 for admission, day 7, and day 21, respectively). The overexpression of miR-155-5p has significantly increased inflammatory cytokine production and promoted COVID-19 progression. We speculated that microRNA-155 facilitates immune inflammation via targeting *SOCS1*, thus establishing its association with disease prognosis.

## 1. Introduction

Understanding host response to SARS-CoV-2 sheds light on viral pathogenesis and improves patient follow-up. The clinical presentation of COVID-19 patients may range from non-specific symptoms to severe systemic findings. The severity of the disease is affected by epigenetic regulators and genetic background. The host-encoded microRNA (miRNA) response to COVID-19 is essential to predicting disease progression [1]. miRNAs are short non-coding RNAs of approximately 22 nucleotides in length involved in regulating gene expression [2]. In this era, miRNAs have been considered highly innovative biomarkers, which can be translated into diagnostic predictive tools and novel therapeutic targets. Several viral infections have been linked with the abnormal expression of numerous miRNAs. Viruses can induce the up-/downregulation of various host miRNAs to evade the host’s immune system. Viral infection-induced changes occur in the expression profile of host miRNAs participate in various signaling pathways, the modulation of host-virus interactions, the regulation of viral infectivity, transmission, and the activation of antiviral immune responses [3]. Most COVID-19 studies address significant variations in clinical manifestations and outcomes. In this context, it is critical to prevent unnecessary treatment of low-risk patients while providing appropriate interventions for patients at high risk of complications [4]. The reliable predictive tools, therefore, are necessary to monitor patient prognosis.

miR-155 has been commonly investigated in the host–pathogen interactions of human viral infections and found to be associated with immune modulation [5]. miR-155 is critical for cell-mediated immune responses and is expressed in B cells, T cells, and macrophages. miR-155 expression is related to pro-inflammatory transcription and induced in response to inflammatory stimuli within hours. Moreover, miR-155 regulates macrophage responses through modulation of cytokine production [6]. The suppressor of cytokine signaling 1 (SOCS1) is one of the major negative regulators of the JAK/STAT pathway and mediates the inhibition of pro-inflammatory cytokines, including tumor necrosis factor-α (TNF-α), interleukin-6 (IL-6), and interferon-γ (IFN-γ). miR-155 has promoted the production of these cytokines by downregulating SOCS1 and significantly modulated inflammatory response. More recently, miR-155 antagomiRs were observed to decrease the production of TNF- α and IL-6 and increase the anti-inflammatory cytokines through increases in SOCS1 [7]. The application of specific antagomiRs against miRNAs involved in the inflammatory process has been proposed to attenuate the cytokine storm and decrease lung damage in COVID-19 patients [8]. Here, we analyzed expression profiles of circulating miR-21, miR-24, miR-122, mir124, miR-126, miR-146, miR-155, miR-200C, miR-196, miR-136, and miR-744 in whole blood samples of hospitalized COVID-19 patients. Since these miRNAs are recognized to be involved in pathways associated with COVID-19, their expression levels have potential value for the diagnosis and severity prediction of inflammatory response in COVID-19. Accordingly, our study was designed to explore the utility of the biomarkers based on disease severity. We aimed to examine the expressions of some host miRNAs and their target genes, which are epigenetic factors affecting the clinical severity of COVID-19. Thus, it was intended to shed insight on the disease’s pathophysiology and identify miRNAs that have predictive value in treatment decisions.

## 2. Materials and Methods

### 2.1. Patients

The study was an observational, prospective case–control study that included 73 patients aged ≥18 years with a positive nasopharyngeal swab PCR test for SARS-CoV-2 during the pandemic between May 2020 and December 2020. Additionally, ten healthy patients were enrolled as controls. The study was conducted according to the guidelines of the Declaration of Helsinki. The protocol was approved by the Clinical Research Ethics Committee Bakırköy Dr. Sadi Konuk Training and Research Hospital (Ethical Code: 2020/143) and registered for Clinical Trials on 2 June 2020 (ClinicalTrials.gov identifier: NCT04411563) [9]. 

According to the severity of disease at admission or throughout hospitalization, the patients were classified into three groups as follows: (1) Moderate: patients who showed evidence of lower respiratory disease during clinical assessment or imaging and who had an oxygen saturation (SpO2) ≥ 94% at room air; (2) Severe: patients who had SpO2 < 94% on room air and required invasive or non-invasive ventilation; (3) Critically ill: patients who had respiratory failure, septic shock, and multiple organ dysfunction [10]. Patients with comorbidities, including diabetes, chronic obstructive pulmonary disease, malignancy, ischemic heart disease, chronic renal failure, and cerebrovascular accident, and those who were receiving immunosuppressive therapy for autoimmune diseases were excluded. Study investigators evaluated their clinical status daily from day 1 to day 21 or until hospital discharge. If the clinical grade of a hospitalized patient changed on a particular day, this was documented and the patient’s group was switched. A final assessment was conducted on day 21 for hospitalized patients or by recalling those discharged.

### 2.2. Data and Sample Collection

All the patient data were obtained from the Hospital Information System called “Probel.” Demographic characteristics included age, gender, weight, and smoking status. Biochemical parameters and radiological images obtained from patients’ medical records included markers of inflammation (C-reactive protein (CRP), ferritin), coagulopathy (d-dimer, fibrinogen, and the international normalized ratio; INR); acute kidney injury (creatinine); acute cardiac injury (troponin); liver failure (aspartate aminotransferase (AST), alanine aminotransferase (ALT), lactate dehydrogenase (LDH), and complete blood counts (CBC)); and computerized chest tomography (CT).

Blood collection and processing were performed based on standardized protocols proposed by the Early Detection Research Network (ERDN) [11]. Blood samples were collected following standard procedure, under fasting conditions and at the same time of day. Special attention was paid to commonly used drugs in hospitalized COVID-19 patients, and blood samples were collected shortly before drug initiation or last dose administration. First blood samples were collected at admission and before treatment with specific therapies for COVID-19. The second and third blood samples were obtained on days 7 and 21 after hospitalization. Whole blood was used for this study, as serum or plasma provides much less miRNA than cells. All blood samples were collected in a RNA Shield Blood Collection Tube (Zymo Research R1150) and transferred to the laboratory within 30 min. Samples were frozen at −80 °C until further handling and analysis. Eleven miRNAs, miR-21, miR-24, miR-122, mir124, miR-126, miR-146, miR-155, miR-200C, miR-196, miR-136, and miR-744, were selected based on the literature search indicating their role in biochemical signaling pathways involved in viral infections and inflammation [3].

### 2.3. Sample Preparation

Two hundred and nineteen whole blood samples collected in RNA Shield Blood Collection tubes from 73 patients were used for miRNA determination. According to the manufacturer’s protocol, RNA was extracted from 200 μL of whole blood using Zymo Research Direct-zol™ RNA Miniprep Plus Kit (Catalog #R2073). Then, RNA was reverse transcribed to cDNA using a miRNA-specific primer. The concentration of RNA isolates was confirmed, and 5–10 ng/μL of RNA were used for cDNA synthesis using a specific primer (MiRXES ID3EAL™ cDNA Synthesis System Code: 1103103 and ID3EAL Individual miRNA RT Primer 1plex 100 Code: 1103114, BioVendor, Czech Republic). The reverse transcriptase reactions were incubated on a Magnetic Induction Cycler (Mic) qPCR system thermocycler at 42 °C/30 m, followed by heat inactivation at 95 °C/5 m [12].

### 2.4. Real-Time qPCR Amplification and Detection

We analyzed the specified miRNAs on the cDNA samples using quantitative real-time PCR (MiRXES ID3EAL Individual miRNA qPCR Assay 100, Code: 1104101 and ID3EAL miRNA qPCR Master Mix 800 Code: 1104204). The cDNA samples were diluted ten times with nuclease-free water, and diluted cDNA templates were pipetted to each PCR reaction well. A total of 20 μL of PCR reaction volume were prepared by combining 10 μL 2× ID3EAL qPCR MasterMix, 5 μL diluted cDNA, 2 μL 10× ID3EAL miRNA qPCR assays, 1 μL ID3EAL individual miRNA RT Primer (Hsa-miR-155-5p: MIMAT0000646, microRNA Seq: UUAAUGCUAAUCGUGAUAGGGGU) and 3 μL nuclease-free water. Real-time PCR amplification was performed in a 96-well plate at 95 °C for 10 min, 40 °C for five min and followed by 40 cycles of 95 °C for 10 s and 60 °C for 30 s [13].

Quantitative PCR was performed on Mic qPCR Cycler, a 4-channel machine (Bio Molecular Systems (BMS), Gold Coast, Australia). The cycle threshold (Ct) values were calculated with Mic RQ (Relative Quantification), software Version 2.10.1. The relative quantification method was used to analyze the differences in miRNA expression of the patient groups compared to the control group. A reference miRNA (miR-U6) was run parallel with target miRNAs to ensure the normalization and validation of variations in sample loading. The efficiency of each miRNA was taken into account in the calculation of expression values. The software also provides the delta–delta Ct method [14], which assumes an efficiency value of 1 for each miRNA used. Accordingly, the CT value of reference miRNA (miR-U6) was subtracted from that of each target miRNA to obtain the ΔCt values of the control and patient samples. The ∆∆Ct value was then obtained by subtracting the ΔCt value of the control sample from that of the patient sample. Since ∆∆Ct values correlate inversely with the amount of template miRNA, fold variations between samples were calculated by the by 2^−∆∆Ct^ method, and the results were stated as “fold change”. A comparison of the 11 miRNAs using initial data of qPCR analysis revealed that miR 155 5p showed significant relevance in the samples of the patients.

### 2.5. Quantitation of SOCS1 Expression

In the literature, it has been previously shown that miR-155 directly regulates the expression of SOCS1, a negative regulator of the macrophage inflammatory response [6,7]. We focused on ***SOCS1*** as the target gene of most significantly differentially expressed miRNA-155 in our patients. For expression analysis of *SOCS1* by RT-qPCR, 5 µg of the RNA sample was reverse transcribed using reverse transcriptase (*Bioline* SensiFAST cDNA Synthesis Kit, BIO-65053) according to the manufacturer’s protocol. Then, with specific primers (Forward Primer: 5′CACGCACTTCCGCACATTC3′, Reverse Primer: 5′TAAGGGCGAAAAAGCAGTTCC3′) for *SOCS1* transcript NM_003745.2, SYBR green-based real-time PCR was performed (SensiFAST SYBR No-ROX Kit, BIO-98005) using a BMS MIC qPCR 4-channel system. The relative expression of mRNA was normalized to that of the reference gene *GAPDH,* and fold variations among patients’ samples were calculated using the 2^−∆∆Ct^ method [14].

### 2.6. Statistical Analysis

Statistical analysis was performed with the NCSS (Number Cruncher Statistical System) 2007 Statistical Software (Utah, USA) package program. The distribution of variables was investigated with the Shapiro–Wilk test of normality to analyze the study data and descriptive statistical methods (mean, standard deviation, median, interquartile range). One-way analysis of variance was used for intergroup comparisons, and Tukey’s multiple comparison test was used for subgroup comparisons of normally distributed variables. For time comparisons of non-parametric variables, the Friedman test was used. Dunn’s multiple comparison test was used for subgroup comparisons, and the Kruskal–Wallis test was used for intergroup comparisons of non-parametric variables. The Chi-square test was applied to compare qualitative data, and the Pearson correlation test was used to determine the relationship between two variables. The results were evaluated at the significance level of *p* < 0.05.

## 3. Results

This study examined the effect of epigenetic factors in three groups of patients with different clinical severity of COVID-19, although there was no comorbid disease. Expression changes of eleven miRNAs associated with immune response pathways to viral infections were analyzed by RT-PCR, and the upregulation of miR-155 was found to be associated with increasing disease severity. Additionally, expression levels of its target gene, SOCS1, were down-regulated and negatively correlated with miR-155 level and disease severity.

### 3.1. Demographic Characteristic and Laboratory Findings of Patients

There was no statistically significant difference between the mean age and gender distribution of the moderate, severe, and critical groups (*p* = 0.279, *p* = 0.257) (Table 1). A statistically significant difference was observed between the distributions of the CT findings of the groups (*p* = 0.0001) A statistically significant difference was observed between the status distributions of the groups 28 days after admission to the hospital (*p* = 0.0001). The mortality rate in the critical group was higher than in the moderate and severe groups. The laboratory findings of the groups on admission to the hospital are summarized in Table 2.

### 3.2. Quantitative RT-PCR Analyses of miR-155-5p and SOCS1 Expression in the Moderate, Severe, and Critical COVID-19 Patients

**Figure 1:**
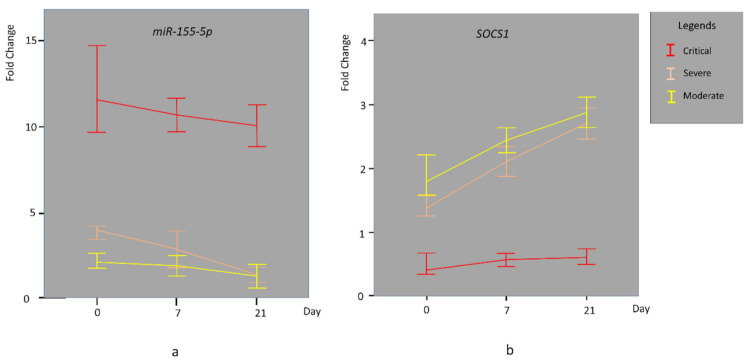
Time-dependent change in expression of miR-155-5p (**a**) and *SOCS1* (**b**) in the moderate, severe, and critical group by 2^−∆∆Ct^-transformed quantification of differential expression. The figure indicates a significant change in the expression of miR-155-5p and *SOCS1* between the day of admission to hospital, day 7, and 21. Data are presented as medians.

**Figure 2:**
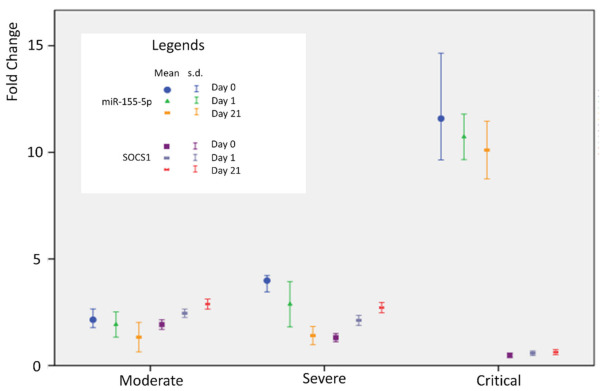
Correlation analysis of the relationship between expression change of miR-155-5p and *SOCS1* variables on admission, day 7, and day 21. Figure shows the relation of the 2^−∆∆Ct^-transformed quantification of the variables and difference expressions and severity of disease.

A statistically significant difference was observed in the relative expression (fold change) of miR-155-5p among the moderate, severe, and critically ill groups (*p* = 0.0001). In addition, the relative expression of miR-155-5p was significantly different between the day of admission to the hospital, day 7, and day 21 (*p* = 0.0001) (Table 3). As shown in Figure 1a patients in the critically ill group had the highest levels of miR-155 expression, indicating that miR-155 expression levels were positively correlated with disease severity. *SOCS1* has been reported as a target of suppression by miR-155. Therefore, *SOCS1* expression was analyzed by RT-qPCR. The relative SOCS1 expression (fold change) decreased with increased disease severity, with the critical group of patients displaying the lowest levels of SOCS1 expression (*p* = 0.0001 for each group) (Table 3, Figure 1b). A statistically significant negative correlation was observed between miR-155-5p values and *SOCS1* values on admission to the hospital, on days 7 and 21 (r = −0.805 *p* = 0.0001, r = −0.940 *p* = 0.0001, r = −0.933, *p* = 0.0001, respectively) (Table 4, Figure 2). The other selected miRNAs indicated different expression patterns in moderate, severe, and critically ill COVID-19 patients. However, none of them could predict the severity of the disease.

## 4. Discussion

This study investigated the relationship between the severity of COVID-19 disease and alterations in the host’s miRNAs based on miRNA-mediated pathways and epigenetic regulation. We found a negative correlation between the relative expression of miR-155-5p and SOCS1 during different stages of the COVID-19 course. Our results suggest that miRNA-155-5p-dependent regulation of SOCS1 expression affects disease severity and regulates responses to viral stimuli. Thus, miR-155 may serve as a predictive tool to classify clinical severity or therapeutic target in COVID-19 patients.

Routine biochemical parameters and chest CT findings present significant insights for disease prognosis [15,16] but cannot fully explain the mechanisms that alleviate or exacerbate pathogenesis and severity of the disease. Although age and comorbid conditions are among the risk factors associated with disease severity and death [17], morbidity and mortality rates in young and non-comorbid individuals suggest underlying genetic and epigenetic mechanisms. Therefore, in COVID-19, research for biomarkers to predict disease severity is still ongoing.

In this research, patients with chronic disorders, such as diabetes, chronic obstructive lung disease, cancer, ischemic heart disease, and chronic renal failure, were excluded from the study to avoid the impact of pre-existing comorbid disorders. In addition, our patient groups were similar in terms of age and gender. Our results also revealed significant differences in biochemical tests, including CRP, pro-calcitonin, D-dimer, fibrinogen, hematological parameters, and chest CT findings, according to the severity of the disease. However, these differences were insufficient to explain the variability in disease severity. To add further insights into the prognosis of SARS-CoV-2 infection, we examined the expression of levels of host miRNAs involved in viral infections and potentially associated with inflammation and respiratory symptoms [18,19,20,21] in 73 hospitalized cases of COVID-19. Accordingly, one of the 11 analyzed host miRNAs, miR-155-5p, was strongly upregulated in the critically ill group. The expression of *SOCS1*, an essential target gene of miR-155-5p, exhibited a negative and robust correlation with miR-155-5p. Increased miR-155-5p levels found in our study align with previous studies that reported that miR-155-5p was significantly upregulated in COVID-19 patients [22,23,24]. Considering the role of SOC1 as a modulator of anti-viral responses, evidence of down-regulated *SOCS1* expression by the overexpression of miR-155-5p in patients with COVID-19 observed in the present study is noteworthy. We also highlighted a significant time-dependent change in miR-155-5p and SOCS1 levels during COVID-19. Among miRNAs, circulating miR-155 (down-regulated or up-regulated) has been considered one of the most promising diagnostic and predictive biomarkers of COVID-19 [25]. In contrast to the literature and our findings, a recent study found reduced expression levels of miR-155 in the serum of COVID-19 patients. This inconsistency may be caused by using serum samples instead of cell samples primarily preferred in other miR-155 studies due to their functions in the cell and enhanced uptake of miR-155 into the cells during the COVID-19 infection and selective miRNA degradation by SARS-CoV-2 [26].

To understand underlying molecular pathology in severe cases of COVID-19, single-gene mutation studies and polymorphism studies [27,28,29] as well as numerous epigenetic investigations have been carried out [30,31,32]. SARS-CoV-2 has been found to uniquely target immune-signaling pathways, such as autophagy and IFN-I signaling, which may explain the prolonged asymptomatic period in COVID-19. Cellular miRNAs induced by SARS-CoV-2 may boost the host immunity and modulate immune evasion for virus survival. It has been demonstrated that the relationship between host miRNAs and SARS-CoV-2 can induce viral pathogenesis by dysregulating antiviral immune responses and signaling pathways, which might lead to worsening complications in comorbid patients with cardiovascular disorders, diabetes, and respiratory problems. In the light of these, miRNAs can be a critical epigenetic modulator that might help design RNA therapeutics to alleviate the complications of COVID-19 [33].

The miR-155, which exhibited significantly different expression levels in patients with varying degrees of disease severity in our study, has a range of known biological functions, including the induction of Toll-like receptor (TLR) activation in monocytes/macrophages and the modulation of TLR signaling, facilitating pro-inflammatory cellular responses and initiating systemic inflammatory responses [34]. A single miRNA has been shown to influence the expression of hundreds of target genes. However, any effect of a single target on its function is unclear. According to exact theories, the function of a single miRNA–mRNA interaction varies depending on the cell type and biological pattern. The crucial role of miR-155-mediated *SOCS1* regulation in specific cellular and biological mechanisms do not eliminate the possible involvement of other miR-155 targets [35]. Studies in larger patient cohorts are needed to understand the effect of MiR-155 and *SOCS1* interaction on the progression of viral infections. These studies will provide knowledge in terms of disease pathophysiology and therapeutic target molecule research.

## 5. Conclusions

Our study group is valuable as it consists of COVID-19 cases of similar age and gender and patients did not have a pre-existing comorbid disease. However, the limited number of patients in the groups is a limitation of the study. Because miRNAs are active epigenetic modulators, results need to be validated in large patient groups. Our results support that host miR-155-5p and *SOCS1* may indeed play a role in the prognosis of COVID-19.

## Figures and Tables

**Table 1:** Characteristics of patients according to the severity of COVID-19 pneumonia (classified as moderate, severe, critically ill).

Variable		Moderate (*n* = 37)	Severe (*n* = 25)	Critically Ill (*n* = 11)	*p*-Value
Age (year)	Mean ± SD	56.05 ± 13.72	59.64 ± 14.84	51.27 ± 16.95	0.279 *
Gender	Female, *n* (%)	18 (48.65)	7 (28.00)	4 (36.36)	0.257 ^+^
	Male, *n* (%)	19 (51.35)	18 (72.00)	7 (63.64)	
Chest CT Findings	Mild, *n* (%)	16 (43.24)	3 (12.00)	1 (9.09)	
	Moderate, *n* (%)	17 (45.95)	7 (28.00)	3 (27.27)	**0.0001** ^+^
	Severe, *n* (%)	4 (10.81)	15 (60.00)	7 (63.64)	
Patient status 28 days after hospital admission	Discharge, *n* (%)	30 (81.08)	8 (32.00)	0 (0.00)	
	Continuing treatment	6 (16.22)	17 (68.00)	8 (72.73)	**0.0001** ^+^
	Mortality, *n* (%)	1 (2.70)	0 (0.00)	3 (27.27)	

* One way ANOVA; ^+^ chi square test; CT: computerized chest tomography. Bold values denote statistical significance at the *p* < 0.05 level.

**Table 2:** Laboratory findings of COVID-19 patients in the moderate, severe, and critical groups on admission to the hospital.

		Moderate (*n*: 37)	Severe (*n*: 25)	Critically Ill (*n*: 11)	*p*-Value
Glucose (mg/dL)	Mean ± SD	134.77 ± 46.97	144.86 ± 63.95	161.75 ± 66.43	0.368 *
Urea (mg/dL)	Mean ± SD	34.81 ± 20.53	38.14 ± 18.74	31.68 ± 12.3	0.614 *
Creatinine (mg/dL)	Mean ± SD	2.57 ± 10.02	0.97 ± 0.31	0.83 ± 0.31	0.325 ^‡^
	Median (IQR)	0.85 (0.66–1.02)	0.94 (0.79–1.13)	0.74 (0.55–1.04)	
eGFR (mL/min/1.7)	Mean ± SD	89.46 ± 26.23	84 ± 24.74	101.64 ± 14.86	0.067 ^‡^
	Median (IQR)	92 (78.5–106)	81 (63–98)	97 (89–116)	
AST (U/L)	Mean ± SD	46.53 ± 32.97	60.68 ± 44.1	27.9 ± 11.49	**0.027** ^‡^
	Median (IQR)	33 (25.4–54.5)	48 (31.7–76)	27 (18–35)	
ALT (U/L)	Mean ± SD	42.69 ± 43.92	60.68 ± 60.56	23.54 ± 13	0.162 ^‡^
	Median (IQR)	26 (15.75–56.5)	46.2 (17–78.5)	19 (12.4–30.5)	
GGT (U/L)	Mean ± SD	98.93 ± 245.24	62.05 ± 52.39	71.2 ± 81.38	0.861 ^‡^
	Median (IQR)	42.25 (25.5–80)	50 (25–76)	38.8 (14.73–119.43)	
LDH (U/L)	Mean ± SD	340.64 ± 180.01	369.2 ± 151.32	321.91 ± 137.81	0.398 ^‡^
	Median (IQR)	282.5 (243–369.5)	353 (244.5–478)	274 (208–472)	
CK (U/L)	Mean ± SD	145.36 ± 145.04	380.91 ± 525.99	141.31 ± 106.76	0.542 ^‡^
	Median (IQR)	95 (42.25–206.75)	119 (45–601)	83 (65–274)	
Lipase (U/L)	Mean ± SD	43.73 ± 39.33	59.59 ± 43.78	36.19 ± 48.77	**0.044** ^‡^
	Median (IQR)	31.98 (15.13–62.03)	49.69 (34.42–79.3)	19.34 (14.29–24)	
Ca (mg/dL)	Mean ± SD	8.79 ± 0.63	8.6 ± 0.63	8.86 ± 0.45	0.375 *
Phosphorus (mg/dL)	Mean ± SD	3.11 ± 0.73	2.9 ± 0.54	3.16 ± 0.96	0.481 *
Magnesium (mg/dL)	Mean ± SD	1.98 ± 0.27	1.91 ± 0.27	1.94 ± 0.24	0.611 *
Ferritin (μg/L)	Mean ± SD	336.54 ± 335.75	539.91 ± 562.68	421.14 ± 432.4	0.367 ^‡^
	Median (IQR)	184.3 (88.15–599.1)	344 (150–820)	214.35 (136.65–589.8)	
CRP (mg/L)	Mean ± SD	71.32 ± 76.58	117.87 ± 97.12	155.61 ± 73.06	**0.003** ^‡^
	Median (IQR)	48.54 (15.58–105.11)	104.1 (32.38–179.37)	123.16 (107–188)	
Procalcitonin (ng/mL)	Mean ± SD	0.16 ± 0.19	0.38 ± 0.65	2.4 ± 0.8	**0.0001** ^‡^
	Median (IQR)	0.08 (0.04–0.2)	0.13 (0.06–0.525)	2.115 (2.03–3.19)	
D-dimer (μg FEU/mL)	Mean ± SD	0.71 ± 0.98	2.25 ± 2.54	2.09 ± 0.48	**0.001** ^‡^
	Median (IQR)	0.34 (0.22–0.55)	1.41 (0.34–4)	2.08 (1.69–2.41)	
PT (s)	Mean ± SD	15.27 ± 4.76	14.49 ± 1.51	18.37 ± 10.01	**0.028** ^‡^
	Median (IQR)	13.75 (12.95–15.35)	14.9 (13.35–15.45)	15.65 (14.48–17.3)	
INR	Mean ± SD	1.21 ± 0.46	1.47 ± 0.84	1.53 ± 1.21	**0.042** ^‡^
	Median (IQR)	1.055 (1.01–1.215)	1.15 (1.03–1.31)	1.245 (1.14–1.38)	
aPTT (s)	Mean ± SD	41.11 ± 15.39	49.6 ± 34.03	39.81 ± 8.26	0.198 ^‡^
	Median (IQR)	35.4 (32.9–39.4)	41.3 (34.4–52.5)	41.2 (31.6–45.4)	
Fibrinogen (mg/dL)	Mean ± SD	518 ± 316.87	469 ± 98	713.88 ± 142.66	**0.002** ^‡^
	Median (IQR)	454 (373–555.5)	467 (398–526)	719 (576.75–838.5)	
Troponin I (ng/mL)	Mean ± SD	46.63 ± 211.57	17.86 ± 27.58	9.09 ± 5.39	0.772 ^‡^
	Median (IQR)	6 (4–13)	7 (3.75–21)	9 (3–13)	
WBC (10^3^/µL)	Mean ± SD	6.97 ± 3.32	7.57 ± 2.87	10.58 ± 2.29	**0.014** *
Hemoglobin (g/dL)	Mean ± SD	12.56 ± 1.84	12.52 ± 1.81	12.96 ± 1.65	0.779 *
Hematocrit (%)	Mean ± SD	37.66 ± 4.26	37.39 ± 5.52	38.99 ± 5.24	0.653 *
Platelet (10^3^/µL)	Mean ± SD	230.25 ± 81.87	256 ± 130	197 ± 42.71	0.224 *
Neutrophil (10^3^/µL)	Mean ± SD	4.91 ± 3.33	5.53 ± 2.9	5.46 ± 2.69	0.321
	Median (IQR)	3.77 (2.4–5.9)	5.1 (3.5–7)	5.43 (3.3–8)	
Lymphocyte	Mean ± SD	1.52 ± 0.82	1.38 ± 0.73	1.11 ± 0.75	**0.015** *
Neu %	Mean ± SD	65.42 ± 14.17	70.04 ± 16.68	68.46 ± 16.82	0.514 *
Lym %	Mean ± SD	25.01 ± 12.34	21.36 ± 14.19	22.45 ± 13.7	0.479 ^‡^
	Median (IQR)	24.1 (15.9–31.2)	21.8 (9.9–27.5)	21.7 (10.3–29.9)	

* One-Way Analysis of Variance; ^‡^ Kruskal–Wallis test; IQR: Interquartile range; Bold values denote statistical significance at the *p* < 0.05 level. eGFR: estimated glomerular filtration rate; AST: aspartate aminotransferase; ALT: alanine aminotransferase; GGT: γ Glutamyltransferase; LDH: lactate dehydrogenase; CK: creatine kinase; Ca: calcium; CRP: C-reactive protein; PT: prothrombin time; INR: international normalized ratio; aPTT: activated partial thromboplastin time; WBC: white blood cell.

**Table 3:** Differential expression of miRNA-155 and its target gene *SOCS1* in the moderate, severe, and critical group. The relative expression of miR-155 and *SOCS1* was determined and expressed as the fold change relative to the control miR-U6 and *G6PDH* gene. Data are presented as medians and interquartile ranges.

			Moderate (*n*: 37)	Severe (*n*: 25)	Critical (*n*: 11)	*p* ^‡^
miR-155-5p	Admission to hospital	Mean ± SD	2.696 ± 2.162	4.748 ± 3.269	11.651 ± 2.281	**0.0001**
		Median (IQR)	2.15 (1.56–2.96)	3.98 (3.04–4.66)	11.527 (9.65–13.55)	
	Day 7	Mean ± SD	1.925 ± 1.784	2.875 ± 2.572	10.656 ± 1.436	**0.0001**
		Median (IQR)	1.25 (1.09–2.01)	2.46 (1.61–2.99)	10.627 (9.67–11.68)	
	Day 21	Mean ± SD	1.33 ± 2.064	1.406 ± 1.039	10.044 ± 1.805	**0.0001**
		Median (IQR)	0.95 (0.31–1.23)	1.09 (0.95–1.81)	9.657 (8.65–10.85)	
		*p* ^†^	**0.0001**	**0.0001**	**0.003**	
SOCS1	Admission to hospital	Mean ± SD	1.921 ± 0.68	1.308 ± 0.468	0.472 ± 0.149	**0.0001**
		Median (IQR)	1.81 (1.41–2.58)	1.39 (1.09–1.7)	0.42 (0.39–0.53)	
	Day 7	Ort ± SS	2.452 ± 0.584	2.119 ± 0.565	0.566 ± 0.15	**0.0001**
		Median (IQR)	2.65 (2.24–2.82)	2.16 (1.88–2.54)	0.584 (0.42–0.7)	
	Day 21	Ort ± SS	2.885 ± 0.71	2.714 ± 0.584	0.616 ± 0.168	**0.0001**
		Median (IQR)	2.98 (2.74–3.18)	2.89 (2.8–3)	0.618 (0.5–0.72)	
		*p* ^†^	**0.0001**	**0.0001**	**0.02**	

^‡^ Kruskal Wallis Test, ^†^ Friedman Test, IQR: Interquartile range. Bold values denote statistical significance at the *p* < 0.05 level.

**Table 4:** Correlations between expression of miR-155-5p and *SOCS1* on days of admission, 7, and 21.

		miR-155-5p on Admission	miR-155-5p 7th Day	miR-155-5p 21st Day
*SOCS1* on admission	r	−0.805		
	*p*	0.0001		
*SOCS1* day 7	r		−0.940	
	*p*		0.0001	
*SOCS1* day 21	r			−0.933
	*p*			0.0001

Pearson Correlation Test.

## Data Availability

If requested, all patient data may be provided.
